# Supplementary material for: Effect of Brown Algae and Lichen Extracts on the SCOBY Microbiome and Kombucha Properties
Source: Foods. 2022 Dec 22;12(1):47. doi: 10.3390/foods12010047 (PMC9818207; doi:10.3390/foods12010047)
Supplement: Supplementary file 1 [file foods-12-00047-s001.zip › foods-2077495-supplementary.pdf]

Supplementary Materials:

Table S1. Chemical composition of extracts of green tea, algae *Fucus vesiculosus* and lichen *Cetraria islandica*, mg/g dry matter.

	**Green tea**	***Fucus vesiculosus***	***Cetraria islandica***
**Carbohydrates**			
Fructose	8.88	0.37	1.70
Glucose	7.99	0.59	18.41
Maltose	0.24	0.00	0.00
Mannose	1.74	0.51	0.00
Raffinose	3.07	0.00	0.00
**Organic acids**			
Acetic	9.55	8.34	11.61
Citric	0.00	0.97	2.41
Fumaric	0.00	0.00	0.13
Isocitric	0.00	0.00	1.14
α-Ketoglutaric	0.00	0.00	1.46
Quinic	27.85	0.00	0.00
Isovaleric	0.39	0.00	0.00
Caprylic	0.21	0.00	0.00
Gluconic	0.28	0.00	0.00
Glucuronic	0.13	0.00	0.00
**Amino acids**			
Alanine	0.00	0.52	0.00
Glutamic acid	0.00	2.47	0.00
Glycine	0.00	0.00	0.14
**Phenolic acids**			
3-Hydroxyphenylacetic acid	0.27	0.00	0.25
4-Coumaric acid	0.00	0.00	18.88
Ferulic acid	0.00	0.00	0.62
Chlorogenic acid	0.16	0.00	0.00
Gallic acid	1.75	0.00	0.00
**Polyols**			
Glycerol	0.10	0.36	0.53
Mannitol	0.37	0.00	0.00
Myo-inositol	4.02	0.00	0.00
**Fatty alcohols**			
Hexadecanol	0.14	0.00	0.00
Fitol	0.12	0.00	0.00
**Flavonoids**			
Catechin	2.24	0.00	0.00

Table S2. Chemical composition of kombucha samples, mg/g dry matter.

	Kombucha sample							
	K		KF		KC		KFC	
	Fermentation day							
	0	12	0	12	0	12	0	12
**Carbohydrates**								
Fructose	0.54	54.18	0.60	5.68	0.56	49.40	0.58	7.02
Glucose	0.69	24.50	0.89	2.84	0.84	23.54	0.83	5.23
Mannose	0.00	0.00	0.00	0.00	0.00	0.00	0.00	1.73
Raffinose	1.03	1.39	1.03	1.31	1.03	1.22	1.02	1.15
Sucrose	943.40	883.1	960.24	926.0	967.37	831.7	941.28	863.7
**Organic acids**								
Acetic	3.91	4.44	3.81	8.94	3.65	5.12	4.09	6.68
Citric	0.00	0.00	0.00	0.56	0.00	0.00	0.00	0.00
Formic	0.00	0.00	0.00	1.21	0.00	2.14	0.00	0.00
Isocitric	0.00	0.00	1.43	0.00	0.89	0.00	0.00	0.00
Gluconic	0.00	1.28	0.00	1.85	0.00	1.38	0.00	1.92
Pyruvic	0.00	3.65	0.00	0.00	0.00	0.00	0.00	0.00
Quinic	1.75	0.67	1.91	0.00	1.84	1.10	1.94	0.00
Isovaleric	0.08	0.00	0.09	0.00	0.09	0.00	0.10	0.00
Caprylic	0.08	0.00	0.06	0.00	0.06	0.00	0.07	0.00
Propionic	0.00	0.00	0.01	0.00	0.00	0.00	0.04	0.00
Orotic	0.00	0.48	0.00	1.08	0.00	0.23	0.00	0.88
**Amino acids**								
Alanine	0.00	0.40	0.00	0.00	0.00	0.38	0.00	0.00
Glycine	0.00	0.20	0.00	0.24	0.00	0.15	0.00	0.00
Lysine	0.00	0.00	0.00	0.00	0.56	0.00	1.22	0.00
**Phenolic acids**								
3-Hydroxy-phenylacetic acid	0.00	0.00	0.00	0.00	0.00	0.00	0.05	0.00
4-Coumaric acid	0.00	0.09	0.00	1.50	0.00	0.00	0.00	1.61
Ferulic acid	0.00	0.22	0.00	0.00	0.00	0.24	0.00	0.26
**Polyols**								
Glycerol	0.22	0.58	0.00	1.34	0.00	0.47	0.00	1.23
Mannitol	0.00	0.00	0.57	0.00	0.00	0.00	0.50	0.00
Myo-inositol	0.27	0.00	0.30	0.00	0.29	0.00	0.31	0.00
